# Prevalence and Correlates of Ischemic ECG Findings among Adults With and Without HIV in Tanzania

**DOI:** 10.5334/gh.1127

**Published:** 2022-06-10

**Authors:** Sainikitha Prattipati, Francis M. Sakita, Tumsifu G. Tarimo, Godfrey L. Kweka, Jerome J. Mlangi, Amedeus V. Maro, Lauren A. Coaxum, Sophie W. Galson, Alexander T. Limkakeng, Anzibert Rugakingira, Sarah J. Urasa, Nwora L. Okeke, Blandina T. Mmbaga, Gerald S. Bloomfield, Julian T. Hertz

**Affiliations:** 1 Duke Global Health Institute, Durham, North Carolina, USA; 2 Kilimanjaro Christian Medical Centre, Moshi, TZ; 3 Kilimanjaro Christian Medical University College, Moshi, TZ; 4 Division of Emergency Medicine, Duke University School of Medicine, Durham, North Carolina, USA; 5 Tanzania Ministry of Health, Community Development, Gender, Elderly and Children. Dodoma, TZ; 6 Department of Infectious Disease, Duke University School of Medicine, Durham, North Carolina, USA; 7 Kilimanjaro Christian Research Institute, Moshi, TZ; 8 Division of Cardiology, Duke University School of Medicine, Durham, North Carolina, USA; 9 Duke Clinical Research Institute, Durham, North Carolina, USA

**Keywords:** ischemic ECG findings, myocardial infarction, HIV-infection, sub-Saharan Africa

## Abstract

**Introduction:**

HIV confers increased risk of myocardial infarction (MI), but there has been little study of ischemic electrocardiogram (ECG) findings among people with HIV in sub-Saharan Africa.

**Objectives:**

To compare the prevalence of ischemic ECG findings among Tanzanians with and without HIV and to identify correlates of ischemic ECG changes among Tanzanians with HIV.

**Methods:**

Consecutive adults presenting for routine HIV care at a Tanzanian clinic were enrolled. Age- and sex-matched HIV-uninfected controls were enrolled from a nearby general clinic. All participants completed a standardized health questionnaire and underwent 12-lead resting ECG testing, which was adjudicated by independent physicians. Prior MI was defined as pathologic Q-waves in contiguous leads, and myocardial ischemia was defined as ST-segment depression or T-wave inversion in contiguous leads. Pearson’s chi-squared test was used to compare the prevalence of ECG findings among those with and without HIV and multivariate logistic regression was performed to identify correlates of prior MI among all participants.

**Results:**

Of 497 participants with HIV and 497 without HIV, 272 (27.8%) were males and mean (sd) age was 45.2(12.0) years. ECG findings suggestive of prior MI (11.1% vs 2.4%, OR 4.97, 95% CI: 2.71–9.89, *p* < 0.001), and myocardial ischemia (18.7% vs 12.1% OR 1.67, 95% CI: 1.18–2.39, *p* = 0.004) were significantly more common among participants with HIV. On multivariate analysis, ECG findings suggestive of prior MI among all participants were associated with HIV infection (OR 4.73, 95% CI: 2.51–9.63, *p* = 0.030) and self-reported family history of MI or stroke (OR 1.96, 95% CI: 1.08–3.46, *p* = 0.023).

**Conclusions:**

There may be a large burden of ischemic heart disease among adults with HIV in Tanzania, and ECG findings suggestive of coronary artery disease are significantly more common among Tanzanians with HIV than those without HIV.

## Introduction

People with HIV (PWH) in high-income settings are at approximately double the risk of atherosclerotic cardiovascular disease (ASCVD) than those without HIV [1]. Reasons for the association between HIV and ASCVDs like myocardial infarction (MI) include chronic inflammation and immune activation, increased susceptibility to insulin resistance and hyperlipidemia, and perhaps antiretroviral therapy [2,3]. Given recent advances in antiretroviral therapy, ASCVD is now a leading cause of death among PWH in high-income settings [4,5,6]. In sub-Saharan Africa (SSA), where the burden of HIV is greatest [7], there has been substantially less study of ASCVDs like MI among PWH [8].

A recent systematic review identified only two studies examining the burden of MI among PWH in SSA [9]. These studies, both conducted within the same cohort in South Africa in 2006-2008, found a very low burden of MI among PWH [10]. A separate, more recent study found low prevalence of coronary artery calcification among adults both with and without HIV in Uganda using cardiac computed tomography, which is potentially suggestive of low risk for MI [11]. To our knowledge, there have been no other studies of the prevalence or predictors of MI among PWH in SSA. Moreover, although multiple studies have established that PWH in high-income settings are at increased risk of MI [12], there are few studies comparing the MI risk among HIV-infected and HIV-uninfected populations in SSA.

Determining the burden of MI in SSA is challenging, particularly given resource constraints in some areas of the region. In Tanzania, for example, access to cardiac biomarker assays, coronary angiography, echocardiography, and specialized cardiac care is extremely limited [13]. Moreover, a growing body of evidence suggests that even in settings where some diagnostic capacity exists, diagnostic testing for MI is uncommon and MI cases are frequently missed [14,15]. One potential approach for describing the burden of prior MI among PWH in resource-limited settings like Tanzania is through electrocardiogram (ECG) screening. ECGs contain a wealth of information, including evidence of prior MI and myocardial ischemia [16]. A number of studies have examined ECG features of adults with and without HIV in SSA, but these studies have primarily investigated ventricular hypertrophy, arrythmias, and the QTc interval [17,18,19,20,21]. One study examined the prevalence of ischemic ECG findings among a cohort of 155 Ugandan adults with HIV and found that 9% of participants had ischemic ECGs which was not significantly different from HIV-uninfected participants [22]. Beyond this study, there is scant data describing the prevalence of ischemic ECG findings among PWH in SSA, and there is no such data from Tanzania.

The purpose of this study was to [1] determine the prevalence of ECG findings suggestive of prior MI and myocardial ischemia among adults with HIV in Tanzania, [2] to compare the prevalence of ischemic ECG findings among Tanzanians with and without HIV, and [3] to identify correlates of ECG findings of prior MI among PWH in Tanzania. Given growing evidence that QTc prolongation is more common among adults with HIV than those without HIV, placing them at increased risk of sudden cardiac death [18,23,24], a secondary aim of this study was to describe the prevalence of QTc prolongation among Tanzanians with and without HIV. To do so, we conducted a prospective observational study in the Kilimanjaro Region of northern Tanzania.

## Methods

### Setting

This study was conducted at two healthcare facilities in Moshi, Tanzania. Participants with HIV were recruited from Majengo Care and Treatment Center (MCTC), a government-funded clinic that provides routine outpatient HIV care, including antiretroviral (ARV) therapy. MCTC cares for approximately 1200 adults, from both the urban center of Moshi as well as surrounding rural districts. HIV-uninfected controls were recruited from the outpatient department (OPD) at Kilimanjaro Christian Medical Centre (KCMC), a hospital located within the same town. The KCMC OPD cares for adults presenting for low-acuity unscheduled care.

### Participant selection

All patients ≥ 18 years of age presenting to MCTC for routine HIV care were eligible for study inclusion; there were no exclusion criteria. Patients were consecutively approached by study staff upon arrival to MCTC and offered enrollment. Participants were recruited from 1 September 2020 until 1 March 2021.

For each enrolled participant with HIV, an HIV-uninfected control was enrolled in a 1:1 manner from the KCMC OPD. HIV-uninfected controls were age- and sex-matched to enrolled participants with HIV, with age-matching defined as age within five years. Controls were excluded from the study if they reported having HIV. Participants were recruited from 1 October 2020 until 1 October 2021.

### Study procedures

After obtaining informed consent, trained research assistants administered a standardized survey, adapted from the WHO STEPS instrument for non-communicable disease [25]. All participants were asked to provide information about their medical comorbidities, medication use, and lifestyle behaviors. Research assistants measured each participant’s blood pressure, height, and weight. A point-of-care glucose was obtained using a glucometer (GlucoPlus Blood Glucose Monitoring System, GlucoPlus, Montreal, Canada), and participants were asked if they had consumed anything earlier in the day to determine whether the measured glucose value represented a fasting or random glucose. A resting twelve-lead ECG was obtained using a tablet-based ECG machine (PADECG, Edan Instruments, Shenzhen, China).

For participants with HIV, data regarding their history of HIV care, including time since diagnosis, current and prior ART use, duration of ART therapy, most recent CD4, and most recent HIV viral load, were collected directly from their medical record at MCTC.

For HIV-uninfected participants, HIV-negative status was confirmed at time of enrollment via Standard Diagnostics Bioline HIV 1/2 assay (Standard Diagnostics, Suwon, Korea). Pre- and post-counseling accompanied all HIV testing, and any positive results were followed-up with subsequent linkage to appropriate follow-up care.

### Study definitions

ECG findings of prior MI and myocardial ischemia were defined according to ECG criteria in the Fourth Universal Definition of MI guidelines [16]. Specifically, prior MI was defined as the presence of pathologic Q waves in at least two contiguous leads; different cutoffs for pathologic Q wave width and depth were used depending on the specific lead, as per universal guidelines [16]. Myocardial ischemia was defined as ST depressions ≥0.5 mm in at least two contiguous leads or T wave inversions ≥1 mm in at least two contiguous leads. The PADECG machine automatically calculates a QTc interval for each ECG using the Bazett formula. Prolonged QTc was defined as QTc interval ≥450 ms for males and ≥460 ms for females, in accordance with international guidelines [26]. Hypertension was defined by participant self-report of hypertension or measured systolic blood pressure ≥140 mmHg or diastolic blood pressure ≥90 mmHg. Diabetes was defined by participant self-report of diabetes or random glucose ≥126 mg/dl or fasting glucose ≥200 mg/dl. Point-of-care glucose was considered a fasting glucose value if the participant reported consuming nothing earlier in the day other than water, and all other point-of-care glucose results were categorized as random glucose values. Obesity was defined as measured body mass index (BMI) ≥30 kg/m^2^. Virologic suppression was defined as most recent HIV RNA viral load < 200 copies/ml. Sedentary lifestyle was defined as participant self-report of <150 minutes of moderately vigorous exercise per week, as per WHO guidelines [27]. Secondary education was defined as self-report of any formal secondary school education. Family history of MI or stroke was defined as self-report of MI or stroke in a first-degree relative. Lifestyle behaviors such as tobacco use, alcohol use, and fruit and vegetable consumption, were also defined by participant self-report.

### ECG interpretation

All ECGs were interpreted by at least two independent physician adjudicators (JTH, ATL, SWG, FMS, AVM, LC). Physician adjudicators were trained in either emergency medicine or cardiology and were blinded to all clinical data other than participant age and sex. Adjudicators were asked to identify the ECG rhythm (sinus, atrial fibrillation, or other) as well as determine the presence of prior MI and/or myocardial ischemia, using the criteria outlined above. In cases of disagreement, a third physician adjudicator served as the tiebreaker. Agreement between adjudicators regarding the presence of myocardial ischemia was excellent (95.2% agreement, κ = 0.818). Although ECG tracings were obtained and shared with the primarily clinical team immediately, formal adjudication by physician adjudicators for study definition purposes was performed on a delayed basis, typically within one week of enrollment. All clinically actionable ECG findings were shared with the primary clinical team.

### Sample size calculation

There were scant data describing the prevalence of ischemic ECG findings in PWH in SSA from which to estimate an appropriate sample size for this analysis. A large study of patients primarily from Europe and North America found that 18.5% of participants with HIV had ECG evidence of prior infarct or ischemia [28]. Assuming the prevalence of ischemic ECG findings would be the same in our study population, a sample size of 473 persons would be needed to determine this proportion with 95% confidence and a 3.5% margin of error.

### Statistical analyses

The primary study outcome was the prevalence of prior MI among participants with and without HIV. Secondary outcomes were the proportion of participants with myocardial ischemia and prolonged automated QTc interval. BMI was calculated directly from measured weight and height. Pearson’s chi-squared test was used to compare the prevalence of prior MI, myocardial ischemia, and prolonged automated QTc interval among participants with and without HIV. Additional statistical analyses were performed to identify covariates of prior MI among all participants. Univariate associations between participant characteristics and prior MI were assessed via Welch’s t-test for continuous variables or Pearson’s chi-squared for categorical variables. Fisher’s exact test was used when expected cell count was <5. Multivariate logistic regression was then performed to identify predictors of prior MI among all participants. Any variable with evidence of possible univariate association with prior MI (*p* < 0.10) was included in the multivariate model; age and sex were also forced into the model. Additional sub-group analyses were performed to identify covariates of prior MI participants using the same approach for univariate and multivariate analyses outlined above. All statistical analyses were performed in the R suite.

### Sensitivity analyses

Despite matching participants with and without HIV by age and sex, statistically significant differences were identified between the two study populations with regards to seven baseline characteristics: secondary education, hypertension, diabetes, current alcohol use, current tobacco use, sedentary lifestyle, and daily fruit and vegetable consumption. In order to assess for possible confounding by these variables, additional sensitivity analyses were performed by excluding matched participants who were discordant with respect to each of these seven variables. For the purposes of identifying discordant pairs for sensitivity analyses, each possible confounding variable was categorized in binary fashion. After excluding matched participants who were discordant with respect to each of these possibly confounding variables, the prevalence of ECG findings suggestive of prior MI and myocardial ischemia among participants with and without HIV was again compared via Pearson’s chi-squared.

### Ethics and Data Availability

This study received ethical approval from the Tanzania National Institute for Medical Research, the ethics committee at Kilimanjaro Christian Medical Centre in Tanzania, and the institutional review board at Duke Health. All participants provided written informed consent prior to enrollment. The results of all study investigations were shared with MCTC and KCMC care teams, and participants with abnormal test results were referred for further care. The data that support the findings of this study are available from the corresponding author, upon reasonable request.

## Results

**Figure 1:**
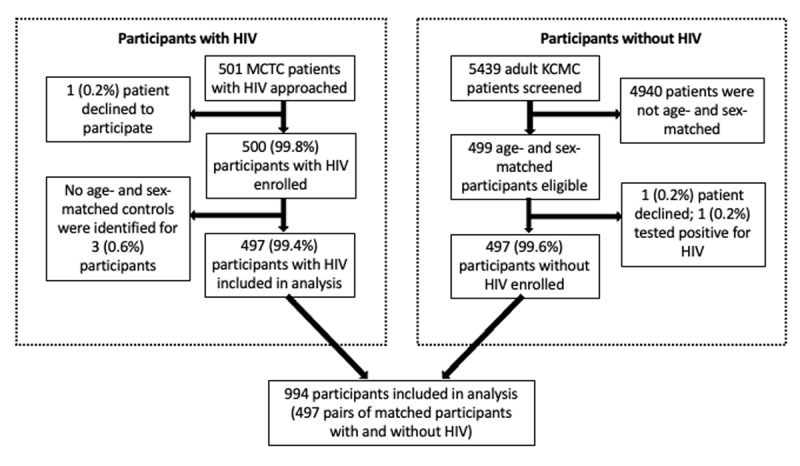
Recruitment of study participants with HIV and without HIV, northern Tanzania, 2020–2021.

Of 501 HIV-infected MCTC patients approached by research staff, 500 (99.8%) consented to participation and were enrolled. In the KCMC OPD, 5439 total patients were screened for potential enrollment as age- and sex-matched HIV-uninfected controls. Of these, 499 were eligible for enrollment. One eligible participant from the KCMC OPD declined to participate and another participant from the KCMC OPD tested positive for HIV and was therefore excluded from the study. This resulted in 497 HIV-uninfected participants. Age- and sex-matched controls were not identified for three MCTC participants prior to study conclusion; therefore, these unmatched participants were excluded from the present analysis (Figure 1).

Participant demographics, medical comorbidities, and lifestyle behaviors of HIV-infected and HIV-uninfected participants are summarized in Table 1. The mean (sd) age of HIV-infected participants was 45.4 (11.3) years, and the mean (sd) age of HIV-uninfected participants was 44.9 (12.7) years (*p* = 0.492). Of HIV-infected participants, 136 (27.4%) were male and 136 (27.4%) of HIV-uninfected participants were also male. Of PWH, 496 (99.8%) were currently receiving ART, and 473 (95.6%) had achieved virologic suppression. Hypertension (OR 0.76, 95% CI: 0.59–0.98, *p* = 0.037), diabetes (OR 0.38, 95% CI: 0.22–0.63, *p* < 0.001), and a sedentary lifestyle (OR 0.74, 95% CI: 0.57–0.95, *p* = 0.021) were less common among HIV-infected participants, whereas participants with HIV were more likely to report daily fruit and vegetable consumption (OR 1.67, 95% CI: 1.10–2.57, *p* = 0.016) and current tobacco use (OR 2.89, 95% CI:1.69–5.18, *p* < 0.001; Table 1). No participant reported a known history of prior MI. The ECG findings of participants are summarized in Table 2. Overall, ischemic ECG findings were significantly more common among participants with HIV than those without HIV, including ECG findings suggestive of prior MI (OR 4.97, 95% CI: 2.71–9.89, *p* < 0.001) and myocardial ischemia (OR 1.67, 95% CI: 1.18–2.39, *p* = 0.004). There was no significant difference in the length of the automated QTc interval among participants with HIV (mean = 419.9, sd = 23.6) compared to participants without HIV (mean = 417.7, sd = 21.5), and between the proportion of participants with prolonged automated QTc among those with and without HIV (OR 1.37, 95% CI: 0.68–2.83, *p* = 0.384).

**Table 1:** Characteristics of adults presenting for routine outpatient HIV care and HIV-uninfected controls matched for sex and age, northern Tanzania, 2020-2021 (N = 994).


	HIV-INFECTED PATIENTS (N = 497)		HIV-UNINFECTED CONTROLS (N = 497)		OR (95% CI)	*P* ^a^
	
	N	(%)	N	(%)		

Male sex	136	(27.4)	136	(27.4)	1.00 (0.77–1.32)	1.000

Age, mean (sd), years	45.4 (11.3)		44.9 (12.7)			0.492

Secondary education	116	(23.3)	262	(52.7)	0.27 (0.21–0.36)	<0.001*

**HIV-related parameters**						

HIV virologic suppression (<200 copies/ml)	473^b^	(95.6)^b^				

CD4, mean (sd), cells/mm^3^	487 (261.7)^c^					

Years since HIV diagnosis, mean (sd)	5.6 (4.2)					

Current antiretroviral therapy						

TDF/lamivudine/dolutegravir	468	(94.2)				

Other	28	(5.6)				

None	1	(0.2)				

History of protease inhibitor exposure	13	(2.6)				

History of abacavir exposure	15	(3.0)				

Duration of antiretroviral therapy, mean (sd), years	5.1 (3.7)					

**Comorbidities**						

Hypertension	172	(34.6)	204	(41.0)	0.76 (0.59–0.98)	0.037*

Diabetes	21	(4.2)	52	(10.5)	0.38 (0.22–0.63)	<0.001*

Self-reported personal history of MI	0	(0)	0	(0)		

Self-reported family history of MI or stroke	96	(19.4)	80	(16.1)	1.25 (0.90–1.73)	0.185

Obese	99	(19.3)	96	(19.3)	1.04 (0.76–1.42)	0.811

**Lifestyle behaviors**						

Current alcohol use	242	(48.7)	193	(38.8)	1.49 (1.16–1.92)	0.002*

Current tobacco use	49	(9.9)	18	(3.6)	2.89 (1.69–5.18)	<0.001*

Sedentary lifestyle	161	(32.4)	196	(39.4)	0.74 (0.57–0.95)	0.021*

Daily fruit and vegetable consumption	62	(12.5)	39	(7.8)	1.67 (1.10–2.57)	0.016*


HIV: Human immunodeficiency virus. TDF: Tenofovir disoproxil fumarateMI: Myocardial infarction. ^a^ *p* values generated from Pearson’s chi-squared for categorical variables and Welch’s t-test for continuous variables. ^b^ Viral load data not available for 2 of 497 participants. ^c^ CD4 data not available for 7 of 497 participants. * *p* < 0.05.

**Table 2:** Electrocardiographic features of adults engaged in HIV care and HIV-uninfected controls, northern Tanzania, 2020–2021 (N = 994).


	HIV-INFECTED PATIENTS (N = 497)		HIV-UNINFECTED CONTROLS (N = 497)		OR (95% CI)	*P* ^a^
	
	N	(%)	N	(%)		

**Rhythm**						

Sinus	497	(100)	496	(99.8)		

Other	0	(0)	1	(0.2)		

**Ischemic findings**						

Prior MI	55	(11.1)	12	(2.4)	4.97 (2.71–9.89)	<0.001*

Myocardial ischemia	93	(18.7)	60	(12.1)	1.67 (1.18–2.39)	0.004*

Either prior MI or myocardial ischemia	136	(27.4)	70	(14.1)	2.29 (1.67–3.18)	<0.001*

**QTc Interval**						

Automated QTc interval, mean (sd)	419.9 (23.6)		417.7 (21.5)			0.132

Prolonged automated QTc interval	19	(3.8)	14	(2.8)	1.37 (0.68–2.83)	0.384


* p < 0.05. ^a^ *p* values generated from Pearson’s chi-squared for categorical variables and Welch’s t-test for continuous variables.

Sensitivity analyses are presented in Table 3. Seven additional analyses were performed, after individually excluding matched participants who were discordant with respect to secondary education, hypertension, diabetes, current tobacco use, current alcohol use, sedentary lifestyle, or daily fruit and vegetable consumption. In each analysis, after the exclusion of discordant participants with regard to each of these variables, the prevalence of ECG findings suggestive of prior MI and myocardial ischemia remained significantly higher among participants with HIV than participants without HIV.

**Table 3:** Sensitivity analyses comparing the prevalence of ECG findings suggestive of myocardial ischemia and prior myocardial infarction among adults with and without HIV in Tanzania.


SENSITIVITY ANALYSIS	PARTICIPANTS WITH HIV, N (%)	PARTICIPANTS WITHOUT HIV, N (%)	*ODDS RATIO (95% CI)*	*P*

Excluding matched participants with discordant education levels	N = 354	N = 354		

Myocardial ischemia	65 (18.5)	37 (10.5)	1.92 (1.25, 3.00)	0.003*

Prior MI	36 (10.3)	7 (2.0)	5.51 (2.55, 13.80)	<0.001*

Excluding matched participants with discordant hypertension	N = 485	N = 485		

Myocardial ischemia	88 (18.1)	58 (12.0)	1.63 (1.14, 2.34)	0.007*

Prior MI	53 (10.9)	12 (2.5)	4.78 (2.60, 9.53)	<0.001*

Excluding matched participants with discordant diabetes	N = 467	N = 467		

Myocardial ischemia	89 (19.1)	56 (12.0)	1.73 (1.20, 2.49)	0.003*

Prior MI	51 (10.9)	11 (2.4)	5.02 (2.67, 10.3)	<0.001*

Excluding matched participants with discordant alcohol use	N = 446	N = 446		

Myocardial ischemia	84 (18.8)	52 (11.7)	1.76 (1.21, 2.57)	0.003*

Prior MI	49 (11.0)	10 (2.2)	5.31 (2.76, 11.30)	<0.001*

Excluding matched participants with discordant tobacco use	N = 464	N = 464		

Myocardial ischemia	86 (18.5)	58 (12.5)	1.59 (1.11, 2.29)	0.011*

Prior MI	47 (10.1)	12 (2.6)	4.20 (2.26, 8.42)	<0.001*

Excluding matched participants with discordant sedentary lifestyle	N = 457	N = 457		

Myocardial ischemia	81 (17.7)	54(11.8)	1.61 (1.11, 2.34)	0.012*

Prior MI	52 (11.4)	12 (2.6)	4.71 (2.56, 9.40)	<0.001*

Excluding matched participants with discordant daily fruit and vegetable consumption	N = 473	N = 473		

Myocardial ischemia	90 (19.0)	59 (12.5)	1.65 (1.16, 2.36)	0.006*

Prior MI	53 (11.2)	11 (2.3)	5.23 (2.80, 10.74)	<0.001*


* *p* < 0.05.

Table 4 presents associations between characteristics of all participants and ECG findings suggestive of prior MI. On univariate analysis, ECG findings suggestive of prior MI among all participants was associated with HIV infection (OR 4.97, 95% CI: 2.71–9.89, *p* < 0.001), family history of MI or stroke (OR 1.95, 95% CI: 1.09–3.36, *p* = 0.026), obesity (OR 0.47, 95% CI: 0.19–0.98, *p* = 0.042), current tobacco use (OR 3.08, 95% CI: 1.45–6.03, *p* = 0.005), and sedentary lifestyle (OR 0.50, 95% CI: 0.27–0.88, *p* = 0.015). On multivariate analysis, ECG findings suggestive of prior MI among all participants was associated with HIV infection (OR 4.73, 95% CI: 2.51–9.63, *p* = 0.030) and family history of MI or stroke (OR 1.96, 95% CI: 1.08–3.46, *p* = 0.023).

**Table 4:** Associations between participant characteristics and ECG evidence of prior myocardial infarction among Tanzanian adults with and without HIV, 2020–2021 (N = 994).


CATEGORICAL VARIABLES	PARTICIPANTS WITH PRIOR MI (N = 67), N (%)	PARTICIPANTS WITHOUT PRIOR MI (N = 927), N (%)	UNADJUSTED UNIVARIATE OR (95% CI)	UNIVARIATE *P^a^*	ADJUSTED MULTIVARIATE OR (95% CI)	MULTIVARIATE *P*

Male sex	28 (41.8)	244 (26.3)	2.01 (1.20–3.33)	0.009*	1.56 (0.87–2.77)	0.132

Secondary education	19 (28.4)	359 (38.7)	0.63 (0.36–1.07)	0.09	1.02 (0.54–1.85)	0.953

HIV-infected	55 (82.1)	442 (47.7)	4.97 (2.71–9.89)	<0.001*	4.73 (2.51–9.63)	<0.001*

Hypertension	25 (37.3)	351 (37.9)	0.98 (0.58–1.63)	0.936		

Self-reported family history of MI or stroke	19 (28.4)	157 (16.9)	1.95 (1.09–3.36)	0.026*	1.96 (1.08–3.46)	0.023*

Obese	7 (10.4)	188 (20.2)	0.47 (0.19–0.98)	0.042*	0.50 (0.20–1.08)	0.104

Current alcohol use	30 (44.8)	405 (43.7)	1.05 (0.63–1.72)	0.86		

Current tobacco use	11 (16.4)	56 (6.0)	3.08 (1.45–6.03)	0.005*	1.64 (0.72–3.51)	0.215

Sedentary lifestyle	15 (22.4)	342 (36.9)	0.50 (0.27–0.88)	0.015*	0.61 (0.32–1.10)	0.112

Daily fruit and vegetable consumption	4 (6.3)	97 (10.5)	0.56 (0.16–1.41)	0.241		

Continuous variables	Participants with prior MI (N=), mean (sd)	Participants without prior MI (N=), mean (sd)		*p*		

Age, years	46.4 (11.9)	45.1 (12.0)		0.372	1.01 (0.99–1.03)	0.469


^a^ Univariate *p* values generated from Pearson’s chi-squared for categorical variables and Welch’s t-test for continuous variables.

Table 5 summarizes associations between characteristics of participants with HIV and ECG findings suggestive of prior MI. On univariate analysis, ECG findings suggestive of prior MI among participants with HIV was associated with male sex (OR 2.48, 95% CI: 1.39–4.41, *p* = 0.002) and current tobacco use (OR 2.31, 95% CI: 1.03–4.82, *p* = 0.044). On multivariate analysis, prior MI among participants with HIV was associated with male sex (OR 2.08, 95% CI: 1.09–3.92, *p* = 0.025) and family history of MI or stroke (OR 2.03, 95% CI: 1.04–3.84, *p* = 0.033).

**Table 5:** Associations between participant characteristics and ECG evidence of prior myocardial infarction among Tanzanian adults with HIV, 2020–2021 (N = 497).


CATEGORICAL VARIABLES	PARTICIPANTS WITH PRIOR MI (N = 55), N (%)	PARTICIPANTS WITHOUT PRIOR MI (N = 442), N (%)	UNADJUSTED UNIVARIATE OR (95% CI)	UNIVARIATE *P^a^*	ADJUSTED MULTIVARIATE OR (95% CI)	MULTIVARIATE *P*

Male sex	25 (45.5%)	111 (25.1%)	2.48 (1.39–4.41)	0.002*	2.08 (1.09–3.92)	0.025*

Secondary education	13 (23.6%)	103 (23.3%)	1.03 (0.51–1.94)	0.939		

HIV virologic suppression (<200 copies/ml)^b^	50 (94.3%)	423 (95.7%)	0.72 (0.23–3.28)	0.626		

History of protease inhibitor exposure	2 (3.6%)	11 (2.5%)	1.57 (0.22–6.15)	0.597		

History of abacavir exposure	4 (7.3%)	11 (2.5%)	3.13 (0.82–9.70)	0.090	3.03 (0.78–9.73)	0.077

Hypertension	22 (40.0%)	150 (33.9%)	1.30 (0.72–2.30)	0.377		

Self-reported family history of MI or stroke	16 (29.1%)	80 (18.1%)	1.86 (0.97–3.45)	0.063	2.03 (1.04–3.84)	0.033*

Obese	6 (10.9%)	93 (21.0%)	0.47 (0.17–1.06)	0.069	0.54 (0.20–1.26)	0.186

Current alcohol use	28 (50.9%)	214 (48.4%)	1.10 (0.63–1.95)	0.730		

Current tobacco use	10 (18.2%)	39 (8.8%)	2.31 (1.03–4.82)	0.044*	1.52 (0.63–3.44)	0.325

Sedentary lifestyle	13 (23.6%)	148 (33.5%)	0.62 (0.31–1.16)	0.141		

Daily fruit and vegetable consumption	4 (7.3%)	58 (13.1%)	0.54 (0.15–1.38)	0.217		

Continuous variables	Participants with prior MI (N = 55), mean (sd)	Participants without prior MI (N = 442), mean (sd)		*p*		

Age, years	46.3 (12.3)	45.3 (11.2)		0.581	1.00 (0.98–1.03)	0.783

CD4, cells/mm^3 c^	445.8 (284.7)	491.9 (258.7)		0.264		

Years since HIV diagnosis	5.3 (4.2)	5.7 (4.3)		0.585		

Duration of antiretroviral therapy	4.7 (3.6)	5.1 (3.7)		0.454		


^a^ Univariate *p* values generated from Pearson’s chi-squared for categorical variables and Welch’s t-test for continuous variables. ^b^ Viral load data not available for 2 of 497 participants. ^c^ CD4 data not available for 7 of 497 participants.

## Discussion

This study is among the first to examine ischemic ECG features among middle-aged PWH in SSA, and to compare the prevalence of ischemic ECG findings among those with and without HIV. We found a high prevalence of ECG findings suggestive of both prior MI and myocardial ischemia among Tanzanians engaged in HIV care, and the prevalence of these ischemic findings was significantly higher among those with HIV than age- and sex-matched controls without HIV. Although nearly all participants had well-controlled HIV, more than one in four had ECGs suggestive of either prior MI or myocardial ischemia. These findings call attention to the need for further study of ischemic heart disease as well as interventions to prevent and treat ischemic heart disease among PWH in SSA.

There are scant data describing the prevalence of ischemic ECG findings among PWH elsewhere in SSA. The 27% prevalence of ischemic ECG findings observed in our study population is substantially higher than the 9% prevalence reported among adults with HIV in Uganda, but might be attributable to higher levels of smoking among PWH in our study [22]. Additional study is needed from across the region to determine whether the high burden of prior MI and myocardial ischemia observed via ECG in PWH in Tanzania is representative of other countries in the region. Notably, the prevalence of ischemic ECG findings observed among PWH in our study is higher than reported in a globally representative low-moderate risk HIV infected population, but similar to what has been reported in older-aged cohorts from Europe and North America [28,29]. The fact that we observed a similar prevalence of ischemic ECG findings in a younger cohort in Tanzania raises the possibility that other factors, such as cardiomyopathies, malnutrition, and poorly-controlled hypertension, may be contributing to the abnormal ECG findings in our study population. In high-income settings, considerable attention has been paid to the rapid increase in cardiovascular disease burden among PWH as HIV care improves and the HIV population ages [30]. There has been less study of the looming burden of cardiovascular disease in SSA, where the world’s largest HIV-infected population is aging rapidly [31]. Our findings suggest that the burden of ischemic heart disease in our setting may already be substantial, particularly among those whose HIV is well-controlled. Importantly, ECG findings of both prior MI and myocardial ischemia were significantly more common among participants with HIV than age- and sex-matched participants without HIV in our study. ECG evidence of prior MI, for example, was approximately five times more common among participants with HIV. This finding is consistent with evidence from high-income settings that HIV is an independent risk factor for MI [1]. Notably, hypertension and diabetes were significantly more prevalent among participants without HIV, making the higher prevalence of ischemic ECG findings among participants with HIV all the more remarkable.

Of particular concern, just over 10% of participants with HIV had ECG evidence of prior MI but none of them were aware of a history of prior MI. This discrepancy suggests that there may be a large burden of silent or unrecognized MI among PWH in Tanzania. Prior study in Tanzania has demonstrated that under-diagnosis of MI is common [15], due in part to inadequate physician training, a lack of diagnostic equipment, and limited patient understanding of the disease [13]. Given these limitations as well as the well-described association between HIV and subclinical atherosclerosis [32,33,34], the high prevalence of ECG findings suggestive of silent or unrecognized MI in our HIV-infected cohort is perhaps unsurprising. In our sub-analysis of participants with HIV, men and patients with a family history of cardiovascular disease were more likely to have ECG findings suggestive of prior MI, so special attention to these groups may be needed in MI screening programs for PWH in Tanzania. Given the preliminary evidence suggestive of a large burden of undiagnosed ischemic heart disease among PWH in our setting, interventions are needed to improve screening for ischemic heart disease and its risk factors. In the absence of routine echocardiography or cardiac magnetic resonance imaging, ECG screening combined with thorough history-taking and physical examination may be a cost-effective approach for screening in resource-limited settings like Tanzania. Given the very low prevalence of prior MI among participants without HIV, our study was underpowered to assess differences in correlates of prior MI among those with and without HIV. A larger study is needed to determine whether there are significant differences in MI risk factors among adults with and without HIV in Tanzania.

Automated QTc prolongation was found in only 4% of participants with HIV, significantly less than what has been reported in similarly-aged adults with HIV in high-income settings [24]. For example, studies in high-income settings have reported a prevalence of 9 to 20% among PWH [35,36,37]. Lower prevalence of prolonged QTc in Tanzania could be due to less frequent use of QTc prolonging medications in this settings. Further study is warranted to determine whether QTc prolongation is indeed rare among PWH elsewhere in SSA and to identify risk factors for prolonged QTc among PWH in the region.

This study had several limitations. First, we enrolled participants with HIV from those who were actively engaged in HIV care; thus, our findings may not be generalizable to patients with undiagnosed HIV or those who are not engaged in care. Men, who tend to be under-represented among those seeking HIV care in SSA [38], were also under-represented in our study. Patients who have already died of HIV-related causes (including MI) would also be underrepresented in our sample. Patients not routinely engaged in care would be less likely to be tested for MI or secondary risk factors. Secondly, we recruited age- and sex-matched participants without HIV from those seeking routine care in the same community, but there were likely other important differences between participants with and without HIV which may have confounded our results. For example, hypertension and diabetes were more common among participants without HIV, which may have led to an underestimation of the effect of HIV on MI risk in our study. Furthermore, because routine non-HIV care in Tanzania generally requires out-of-pocket charges, participants without HIV may have been more socioeconomically advantaged than those with HIV [39,40]. Notably, participants without HIV had higher levels of education attainment at baseline (Table 1), and since education is associated with different lifestyle behaviors and health outcomes, it could explain the lower rates of ischemic ECG findings in our participants without HIV. Importantly, although several differences did exist between participants with and without HIV, account for potential confounders individually did not substantially attenuate the observed association between HIV and ischemic ECG findings in our sensitivity analyses. Thirdly, although we used universal guidelines to define ECG evidence of myocardial ischemia and prior MI [16], ECG findings such as Q waves have limited sensitivity (66–90%) and specificity (85%–90%) for detecting ischemic heart disease when compared to more advanced modalities such as cardiac magnetic resonance imaging [40,41]. Unfortunately, given resource limitations at our study site, obtaining cardiac magnetic resonance imaging, echocardiography, and coronary angiography for our participants was not possible. As access to these modalities improves in Tanzania, additional study will be needed to further characterize ischemic heart disease in this population. Nevertheless, even in the absence of known prior MI, pathologic Q waves have been shown to be strongly predictive of future MI and death [42], highlighting the need for interventions to prevent future cardiovascular events in our study population. Furthermore, we only had access to participants’ most recent CD4 and viral load data; analysis of longitudinal data including CD4 nadir and cumulative viral load may have identified additional predictors of ischemic ECG findings. Finally, our study may have been under-powered to identify all possible predictors of prior MI among study participants. We observed an association between abacavir exposure and prior MI, for instance, and this association may have been statistically significant with a larger sample size.

## Conclusion

In conclusion, among a cohort of Tanzanian adults engaged in HIV care, ECG findings suggestive of prior MI and myocardial ischemia were common. Ischemic ECG findings were significantly more common among adults with HIV than age- and sex-matched adults without HIV. Further study is needed to describe the burden of ischemic heart disease among PWH in SSA, and interventions are needed to improve prevention, screening, and care for MI among Tanzanians with HIV.
